# Undermined Skin Lesion Emerged as Tubercular Meningitis: A Case Report

**DOI:** 10.31729/jnma.8079

**Published:** 2023-03-31

**Authors:** Dhirendra Yadav, Javed Ahmad Khan

**Affiliations:** 1 Patan Academy of Health Sciences, Lagankhel, Lalitpur, Nepal; 2 Department of Internal Medicine, Kapilvastu District Hospital, Taulihawa, Kapilvastu, Nepal

**Keywords:** *case reports*, *lupus vulgaris*, *meningitis*, *skin*, *tuberculosis*

## Abstract

Cutaneous tuberculosis is a rare type of extrapulmonary tuberculosis and it is uncommon even in places where tuberculosis is widespread. A 32-year-old female presented with fever and headache along with prior history of an ulcer in her leg which was treated as cellulitis at another centre. The neck rigidity, and the Kernig and Budzinski sign were also positive. There were also features of increased intracranial pressure. The non-contrast computed tomography showed bilateral hydrocephalus and hypodense areas. She was managed for increased intracranial pressure and anti-tubercular therapy for disseminated tuberculosis. Biopsy of non-healing wounds should be checked for lupus vulgaris.

## INTRODUCTION

Cutaneous tuberculosis (CTB) is caused by Mycobacterium tuberculosis, Mycobacterium bovis, and sporadically by the Calmette-Guerin bacillus.^1^ The combination of several elements, including the infection site and the host's immunity, results in various clinical symptoms. One percent to five percent of the approximately 10 million cases of extrapulmonary tuberculosis (TB) in the world result in tuberculous meningitis (TBM).^2^ A tuberculous chancre is the consequence of direct external tubercle bacilli inoculation of the skin or mucous membranes.^2,3^ there are very few cases reported where the patient has lupus vulgaris and cerebral tuberculosis but no evidence of pulmonary tuberculosis. Here we present a case of a 32-years-female who presented with skin tuberculosis.

## CASE REPORT

A 32-years-female presented to the emergency department with a history of fever and headache for past 3 weeks. An ulcer on her left leg was also seen few weeks prior to these complaints. The fever was insidious on onset associated with diaphoresis and an evening rise in temperature. The headache was generalised with moderate to severe intensity of piercing quality and was partly relieved by medicines. She was shifted to Department of Internal Medicine from ER for symptomatic management with pantoprazole 40 mg, and flexon 400+500 mg. The symptoms reappeared along with neck rigidity which was not associated with trauma. There was also the history of less conversation with the family members which was usually irrelevant in nature. Drowsiness along with misidentifying the things (perceived bucket as a helmet) was also present. There was no history of shortness of breath, cough, chest pain, diarrhoea or constipation, burning micturition, hair fall, rashes, joint pain, or myalgia. The ulcer was present on the left leg which suddenly appeared and grew in size. It started initially as swelling and redness. Incision and drainage were done for it and was treated as cellulitis with an abscess. There was no history of insect bites or any trauma.

There was also a history of decreased hearing in right ear associated with ear discharge since childhood. On examination, the general condition was ill-looking. She was oriented to time and place and the person with a GCS of 10/15 (E4V4M6). The pulse rate was 110 beats per minute, the respiratory rate was 20 breaths/min, the temperature was 38.33°C and SpO_2_ was 95% in the room air. On local examination, the ulcer was 11 cm, undermined with indurated margin and pink base. There was neck rigidity with positive kerning and a Budzinski sign. On central nervous system (CNS) examination, the higher mental function was conferred, comprehensive with decreased alertness, and abnormal memory. Motor and sensory examinations were bilaterally normal. The plantar reflex was upgoing in the right (Babinsky positive) while it was downgoing in the left leg. There was nystagmus present with the impaired cerebellar examination (dysdiadokinesia, heal shin). All cranial nerve's examination was intact.

**Figure 1:**
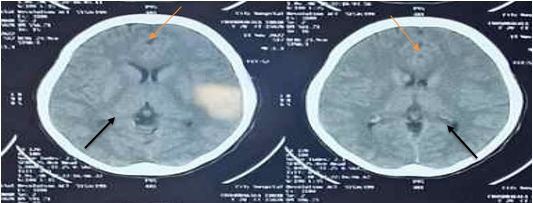
Non-contrast computed tomography showing the features of hydrocephalus (Size of both temporal horns is greater than 2 cm) of the ventricles (Black Arrow) and bilateral temporal showing cerebral infarct (Orange Arrow).

Arterial blood gas was done in which PH was 7.43, PCO_2_ was 36, PO_2_ was 90 millimetres of mercury (mm of Hg) and HCO_3_ was 24.2 millimole per litre (mm/l). The chest X-ray was normal. Also, there was no productive sputum for which Acid-fast Bacilli, as well as Xene Expert, could not be checked. On cerebrospinal fluid (CSF) analysis, it showed a cobweb/spider web in physical appearance, raised total protein, decreased gluose level and raised lymphocyte count, which all pointed towards tubercular meningitis. On the non-contrast computed tomography (NCCT) head, thickening of meninges in basal cistern, and periventricular oedema. The bilateral ventricle shows a feature suggestive of hydrocephalus (Figure 1).

The ECG showed normal sinus rhythm with a normal axis and no left ventricular hypertrophy. A neurosurgery consultation was done which advised gradual lumbar puncture. Also, a biopsy was taken from the ulcer which showed a highly positive gene expert. The serology was non-reactive.

She was given 3% normal saline for increased intracranial pressure (ICP) and started on antitubercular therapy as per national directly observed treatment shortcourse (DOTS) protocolalone with dexamethasone 8 mg thrice daily and ceftriaxone 2 g intravenous twice daily. Her condition improved after 2 weeks of hospital stay and she was improving on the follow-up.

## DISCUSSION

Cutaneous tuberculosis is nearly always caused by *Mycobacterium tuberculosis,* the tubercle bacillus. Cutaneous tuberculosis can develop as a result of the extension of an underlying infective focus into the skin, direct inoculation of tubercle bacilli into the skin, bloodstream spread, or any of these three.^3^ *Mycobacterium tuberculosis* is a non-motile, non-sporulated, unencapsulated bacillus that is straight or slightly bent (rod-shaped), measuring from 1 to 10 micrometre long by 0.2 to 0.6 micrometre wide.^1^ Its most notable characteristic is that it is stained red by fuchsin and does not discolour when exposed to alcohol and acid (acid-fast bacillus).^2^ Due to the high lipid content of its cellular wall, it is resistant to the action of chemical agents but vulnerable to the action of physical forces (heat and ultraviolet radiation).^4^

Central nervous system involvement is a factor in tuberculous meningitis. Initial signs could include headaches and alterations in behaviour which was also seen in this case. Other symptoms include vomiting, a stiff neck, fever, and headaches. Older kids and adults may experience symptoms that develop from irritability to bewilderment, drowsiness, and stupor, possibly culminating in a coma.^5^ In this case, the patient was not in a coma which may be due to timely health-seeking behaviour to a tertiary centre. Seizures, hydrocephalus (fluid buildup in the brain cavity), deafness, mental retardation, hemiparesis (paralysis of one side of the body), and other neurological abnormalities can result from this illness if left untreated.^2,4,5^ Lupus vulgaris has been and remains the most common form of cutaneous tuberculosis. Cutaneous manifestations of disseminated tuberculosis are unusual, being seen in less than 0.5% of cases.^6^ Measles or another major fever infection may be followed by a rare type of lupus vulgaris. A single focus of lupus vulgaris creates a window of immune deficit that leads to acute sickness, which causes TB to spread hematogenously.^7^ A case reported in India showed a tubercular lesion over the cheek without any dissemination and systemic symptoms.^8^ But here in this case the tubercular lesion was on the foot and dissemination to the central nervous system. Another study from India showed the appearance of skin lesions along with pulmonary tuberculosis,^9^ whereas in this case there was no underlying pulmonary tuberculosis.

There are very few cases reported where a person having the presentation of cerebral tuberculosis has preceding skin tuberculosis with no evidence of pulmonary involvement. Individuals with TBM frequently exhibit specific neurological impairments, behavioural problems, and changes in awareness in addition to the usual meningitis symptoms of fever, headache, and meningismus (stiff neck). Adenosine is permanently deaminated by ADA, a key enzyme in the purine metabolism, turning it into inosine. The presence of ADA is thought to be a sign of cell-mediated immunity because it is primarily linked to lymphocyte proliferation and differentiation.^4^ The standard treatment plan for CNS tuberculosis consists of an initial 2-month induction therapy regimen with isoniazid, rifampin, pyrazinamide, and ethambutol, followed by another 7-10 months of isoniazid and rifampin as maintenance therapy for an isolate that is responsive to these drugs.^4,7^

Extrapulmonary tuberculosis lacks the traditional clinical symptoms and imaging diagnostic, making it simple to make a false diagnosis and postpone treatment. The epithelioid granuloma with central caseating necrosis is the distinguishing feature of disseminated and extrapulmonary TB histopathology, and the diagnosis is made based on the discovery of acid-fast bacilli in a smear or culture and/or the presence of caseous granulomas in a tissue specimen. However, TB bacilli are hardly ever observed. The diagnosis of this entity, which is easily controlled medically but can be fatal if untreated, requires a high level of clinical suspicion.
